# Coevolutionary theory of hosts and parasites

**DOI:** 10.1111/jeb.13981

**Published:** 2022-01-30

**Authors:** Lydia J. Buckingham, Ben Ashby

**Affiliations:** ^1^ 1555 Department of Mathematical Sciences University of Bath Bath UK; ^2^ 1555 Milner Centre for Evolution University of Bath Bath UK

**Keywords:** coevolution, host, infectivity, mathematical modelling, parasite, pathogen, red queen, resistance, theory

## Abstract

Host and parasite evolution are closely intertwined, with selection for adaptations and counter‐adaptations forming a coevolutionary feedback loop. Coevolutionary dynamics are often difficult to intuit due to these feedbacks and are hard to demonstrate empirically in most systems. Theoretical models have therefore played a crucial role in shaping our understanding of host–parasite coevolution. Theoretical models vary widely in their assumptions, approaches and aims, and such variety makes it difficult, especially for non‐theoreticians and those new to the field, to: (1) understand how model approaches relate to one another; (2) identify key modelling assumptions; (3) determine how model assumptions relate to biological systems; and (4) reconcile the results of different models with contrasting assumptions. In this review, we identify important model features, highlight key results and predictions and describe how these pertain to model assumptions. We carry out a literature survey of theoretical studies published since the 1950s (*n* = 219 papers) to support our analysis. We identify two particularly important features of models that tend to have a significant qualitative impact on the outcome of host–parasite coevolution: population dynamics and the genetic basis of infection. We also highlight the importance of other modelling features, such as stochasticity and whether time proceeds continuously or in discrete steps, that have received less attention but can drastically alter coevolutionary dynamics. We finish by summarizing recent developments in the field, specifically the trend towards greater model complexity, and discuss likely future directions for research.

## Graphical abstract

Host‐parasite coevolution is a reciprocal process of adaptations and counter‐adaptations. When a parasite evolves to have heightened infectivity, its host may respond by evolving to have heightened resistance.

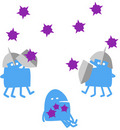

## 1 INTRODUCTION

A wide variety of host defences to parasitism exist in nature, from immune defences such as resistance and tolerance to behavioural defences such as social avoidance and mate choice. These defences exist alongside a plethora of parasitic characteristics (e.g. virulent, avirulent, chronic, acute), life cycles (e.g. one host, multiple hosts) and transmission mechanisms (e.g. airborne, environmental, social, sexual). To explain this diversity requires consideration of how different host and parasite traits have coevolved in response to one another. Host–parasite coevolution consists of adaptation by hosts, to avoid or tolerate infection, and reciprocal counter‐adaptation by parasites, attempting to evade or overcome host defences. This process has been observed across a variety of taxa, including viral and bacterial parasites of animals (Decaestecker et al., 2007; Shim & Galvani, 2009); bacterial, fungal and animal parasites of plants (Frank, 1993a; Iseki et al., 2011; Stahl et al., 1999); and viral parasites of bacteria (Buckling & Rainey, 2002; Koskella & Brockhurst, 2014). Given the ubiquity of parasites throughout the natural world and the severe impact that they often have on host fitness, coevolution is likely to play a major role in a wide range of fundamental biological phenomena. For example, host–parasite coevolution has been implicated in: the evolution of innate and adaptive immune systems (Mayer et al., 2016); the generation and maintenance of genetic diversity within (Altermatt & Ebert, 2008; Penman et al., 2013) and between populations (Kaltz & Shykoff, 1998; Thompson, 1994; Thrall & Burdon, 2002) and through time (Decaestecker et al., 2007; Dybdahl & Lively, 1998; Hall et al., 2011); the evolution of sex (Bell, 1982; Hamilton, 1980; Lively, 2010); sexual selection (Ashby & Boots, 2015; Hamilton & Zuk, 1982); sociality (Ashby & Farine, 2020; Bonds et al., 2005; Prado et al., 2009); and brood parasitism (Servedio & Lande, 2003).

Mathematical modelling has been crucial for developing our understanding of the causes and consequences of host–parasite coevolution, resulting in a rich body of theoretical literature spanning the last 70 years. Haldane's remarks about the potential impact of infectious diseases on natural selection (Haldane, 1949), combined with the discovery of complementary genes for resistance and infectivity in flax and flax rust (Flor, 1956), inspired the first population genetic models (Jayakar, 1970; Leonard, 1977; Mode, 1958; Yu, 1972). Early models considered coevolution at one or two loci, in haploid or diploid hosts, with only frequency‐dependent selection and no epidemiological dynamics. Despite their simplicity, these initial forays into coevolutionary modelling demonstrated the potential for negative frequency‐dependent selection (where the fitness advantage of a trait decreases as it becomes more common), leading to cycling in allele frequencies in both hosts and parasites. Cyclical dynamics were later seized upon in the form of the Red Queen Hypothesis (Bell, 1982) as a solution to the evolutionary enigma of sex (why does sex exist if asexual reproduction is more efficient due to the so‐called ‘twofold cost’ of males (Maynard Smith, 1978)?). Following the seminal theoretical work of Hamilton showing that parasites could indeed select for sex (Hamilton, 1980), there was a surge in interest in modelling host–parasite coevolution, especially in the context of sex and recombination (reviewed in Lively, 2010).

During the 1990s, advances in analytic techniques, such as the development of adaptive dynamics (Hofbauer & Sigmund, 1990), along with increased computing power, enabled modelling of more complex biological scenarios. Crucially, the scope of modelling greatly expanded to consider the genetic basis of infection (Frank, 1993a; Parker, 1994), quantitative traits (Doebeli, 1996; Frank, 1993b; Sasaki & Godfray, 1999), spatial structure (Frank, 1991b; Gandon et al., 1996; Nuismer et al., 1999) and epidemiological dynamics (Frank, 1991b; Gandon et al., 1996). In the twenty‐first century, the field grew still further to encompass an even wider range of scenarios, including more complex infection genetics (Agrawal & Lively, 2002, 2003; Weinberger et al., 2012), superinfection (Gandon et al., 2006; Haven & Park, 2013), vector‐borne pathogens (Koella & Boiëte, 2003), a greater variety of host defence mechanisms (Iranzo et al., 2015) and the role of coevolution in host sociality (Bonds et al., 2005) and mating behaviour (Ashby, 2020a; Ashby & Boots, 2015; Wardlaw & Agrawal, 2019). In recent years, studies have also emphasized the importance of eco‐evolutionary feedbacks by directly comparing equivalent models which include and exclude population dynamics, showing that they qualitatively change the dynamics of host–parasite coevolution (Ashby et al., 2019; MacPherson & Otto, 2018).

The diversity of topics covered in the literature is mirrored by the diversity of approaches taken. Some models are based upon population genetics, whereas others use quantitative genetics; some include population dynamics and eco‐evolutionary feedback loops whereas others do not; assumptions about mutation rates and standing genetic variation differ; different levels of specificity may be worked into the infection genetics; discrete or continuous time may be used to represent non‐overlapping or overlapping generations; models may operate at the level of individuals or populations; the dynamics may be deterministic or stochastic; and results may be obtained analytically, numerically or through simulations. Such a wide range of approaches has led to many advances but also causes difficulties when reconciling predictions or when deciding upon which type of model should be used under which circumstances. This can be especially difficult for newcomers to the field or for those who lack a mathematical background, as the link between model assumptions and results is often unclear.

In this review, we first summarize different types of coevolutionary dynamics and the methods used to study them theoretically. We then discuss significant features of models of host–parasite coevolution and synthesize key predictions about resistance‐infectivity coevolution. Our literature survey reveals that modelling assumptions regarding population dynamics and infection genetics are particularly important. For example, population dynamics typically dampen or reduce the likelihood of fluctuating selection dynamics and increase the incidence of polymorphism. Meanwhile, highly specific infection genetics often lead to rapid fluctuating selection, whereas variation in specificity often lead to stable polymorphism but may also produce fluctuating selection over longer timescales. We also consider the effects of other, less well‐studied features, such as stochasticity and how time is modelled (see Box 1). Finally, we highlight recent advances in eco‐evolutionary theory and discuss future directions for research. Due to limitations on space, we restrict ourselves to overviews of a few key topics. Many of these topics are discussed in more detail in the other sources, including a recent book on the subject, *Introduction to Coevolutionary Theory* (Nuismer, 2017).

BOX 1 **Summary of modelling assumptions and their qualitative effects on coevolution**

**Genetic Structure** describes the underlying genetics which control the traits. Diploidy (as opposed to haploidy) has been found to reduce the incidence of cycling, makes local adaptation more likely and favours assortative mating.

**Infection Genetics** describe which parasites can infect which hosts and to what extent. Highly specific infection genetics often produce rapid fluctuating selection, whereas variation in specialism can produce slower cycles and lead to stable polymorphism.

**Pleiotropy & Trade‐offs** tell us how changes in one trait can affect other traits. Diminishing fitness returns (accelerating trade‐offs) typically favour stable monomorphism. Linear or weakly increasing fitness returns (decelerating trade‐offs) are more likely to lead to evolutionary branching and stable polymorphism.

**Population Dynamics** govern changes in population densities. Their inclusion often increases the likelihood of stable polymorphism, but they also tend to dampen oscillations in allele frequencies or make such oscillations less likely.

**Time** may be modelled as proceeding in discrete steps or may be continuous. Continuous time models may generate damped cycles where discrete time models generate stable cycles.

**Stochasticity** describes random effects. It may cause alleles to reach fixation or cause fluctuating selection to persist when deterministic cycles are damped.

**Spatial Structure** assumes interactions are based on proximity rather than the population being well‐mixed. This generally leads to greater host resistance and lower parasite infectivity; it also makes fluctuating selection more likely. Environmental heterogeneity promotes generalism in hosts and parasites and often increases polymorphism.

## 2 COEVOLUTIONARY DYNAMICS

When analysing models of host–parasite coevolution, theoreticians are often interested in how variation in model parameters (which typically relate to some biological or environmental characteristics or processes) causes quantitative or qualitative changes in evolutionary outcomes. For example, under what conditions do resistance or infectivity increase (quantitative) and when do populations evolve to stable monomorphic or polymorphic equilibria, or exhibit directional or fluctuating selection (qualitative)? From a mathematical perspective, quantitatively different outcomes usually correspond to a change in the *position* of an equilibrium (e.g. the frequency of a resistance allele in the host population or the mortality rate from infection), whereas qualitatively different outcomes correspond to a change in the *stability* of a model (e.g. a stable equilibrium or oscillations). In this section, we outline the various qualitative outcomes one may expect to observe in models of host–parasite coevolution.

Note that coevolution may also lead to the extinction of one or both populations due to, for example, directional selection (Haraguchi & Sasaki, 1996), environmental changes (Wright et al., 2016), stochasticity (Schenk et al., 2020) or competition with other populations (Frank, 1994b). It is possible for a host to drive the parasite extinct (Best, 2018), or vice versa (Seppälä et al., 2020), and once one population is driven extinct, the system can no longer exhibit coevolution.

### 2.1 Stable equilibria

**FIGURE 1:**
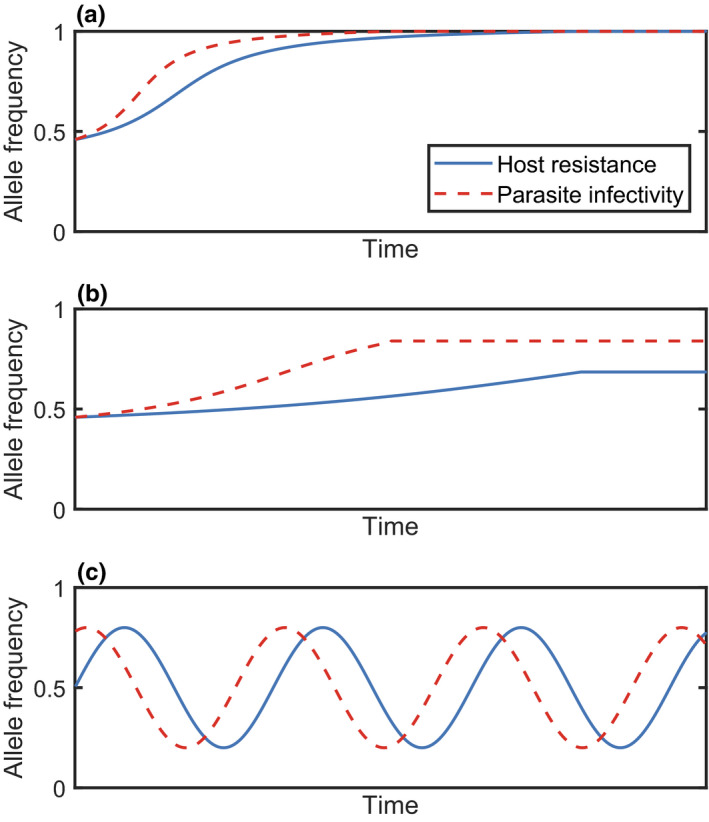
Example time series showing changes in frequencies of host resistance and parasite infectivity alleles. The dynamics shown are (a) directional selection leading to stable monomorphism (alleles go to fixation), (b) stable polymorphism (alleles do not go to fixation but tend to stable frequencies) and (c) fluctuating selection (allele frequencies oscillate over time)

The simplest non‐trivial evolutionary outcome is *stable monomorphism*, where a single type prevails within a population. For example, the host may evolve to a particular level of resistance and the parasite to a particular level of infectivity (Figure 1a). In a quantitative genetic framework (see ‘Genetic Structure’), where traits are continuous, stable monomorphism is usually referred to as an *evolutionarily singular strategy* (ESS) (Maynard Smith, 1972), or a *continuously stable strategy* (CSS) if it is also convergence stable (i.e. it can be approached by small mutations) (Eshel, 1983). Although a single type may be optimal, it is possible for variation to be maintained due to a mutation‐selection balance. Alternatively, one or both populations may exhibit *stable polymorphism*, where two or more types coexist at equilibrium within a population. Stable polymorphism occurs when different alleles at a given locus coexist in the population (Figure 1b) (Ashby et al., 2019; Sasaki, 2000; Tellier & Brown, 2007b), or when disruptive selection leads to a branching process, creating two sub‐populations with different trait values (Best et al., 2009).

### 2.2 Directional selection

Directional selection occurs when there is a continual increase or decrease in traits such as resistance or infectivity (Figure 1). Such dynamics may produce a coevolutionary *arms race*, leading to trait escalation in both species (Dawkins & Krebs, 1979). For example, bacteria and phage often exhibit escalatory arms races under laboratory conditions (Buckling & Rainey, 2002) and plant–pathogen coevolution has led to an escalatory series of defence (e.g. R genes, pathogen‐associated molecular pattern (PAMP) recognition, effector‐triggered immunity) and counter‐defence (e.g. avirulence genes, effector‐triggered susceptibility, toxin production) mechanisms (Jones & Dangl, 2006). Directional selection may also take the form of de‐escalation (Sasaki & Godfray, 1999).

Unlike stable monomorphism and polymorphism, directional selection is a dynamic outcome of coevolution. In principle, directional selection may continue indefinitely, for example when resistance or infectivity increases without bound (Lopez Pascua et al., 2014) or if parasites ‘chase’ hosts through phenotype space without cycling back to previous phenotypes. Directional selection may therefore provide an explanation for the existence of extreme attack and defence traits in parasites and their hosts (Iseki et al., 2011). However, directional selection cannot necessarily be maintained indefinitely due to escalating fitness costs and physiological constraints. As such, periods of directional selection may eventually give way to stable monomorphism, polymorphism or fluctuating selection (Ashby & Boots, 2017; Tellier et al., 2014; Figure 1). For example, Hall et al. (2011) found that although coevolving bacteria and phage initially exhibit directional selection, this eventually gives way to fluctuating selection.

### 2.3 Fluctuating selection

Fluctuating selection (also referred to as coevolutionary cycling or Red Queen Dynamics) occurs when either the direction of selection changes, resulting in host and parasite traits varying non‐monotonically through time, or when there is no stable distribution of genotypes, resulting in oscillations in allele frequencies (Figure 1c). These dynamics tend to be driven by negative frequency‐dependent selection (i.e. rare advantage) (Dybdahl & Lively, 1998; Hamilton, 1980), fitness costs (Ashby & Boots, 2017; Sasaki, 2000), environmental fluctuations (Mostowy & Engelstädter, 2011) or stochasticity (Stephan & Tellier, 2020). Empirically, fluctuating selection has been observed using time‐shift experiments (Gaba & Ebert, 2009), where hosts from one time point are exposed to parasites from another, leading to oscillatory patterns of resistance and infectivity (Decaestecker et al., 2007; Hall et al., 2011; Jokela et al., 2009; Stahl et al., 1999). However, fluctuating selection can be difficult to detect as it may appear as directional selection over short time scales (Figure 1c), where periods of escalation alternate with periods of de‐escalation (Gaba & Ebert, 2009; Gandon et al., 2008). Fluctuating selection may also occur at different levels simultaneously. For example, fluctuations may occur in the range of hosts which parasites can infect (specialism‐generalism) while the specific hosts that they can infect vary as well (Ashby & Boots, 2017). Fluctuating selection may also occur in the short term in the form of damped cycles, which diminish in amplitude and eventually tend towards stable polymorphism (Ashby & Gupta, 2014; MacPherson & Otto, 2018).

Fluctuating selection is closely associated with the Red Queen Hypothesis for sex (Bell, 1982; Lively, 2010), which argues that sexual reproduction is maintained despite being less efficient than asexual reproduction due to coevolution with parasites. This is because sex generates diverse offspring, which allows sexually reproducing hosts to adapt more rapidly than asexual hosts when targeted by coevolving parasites. Understanding when host–parasite coevolution leads to fluctuating selection and investigating how the nature of the oscillatory dynamics (e.g. amplitude, frequency) selects for sex have therefore been major drivers of theoretical research on host–parasite coevolution (reviewed in Ashby & King, 2015; Lively, 2010).

## 3 THEORETICAL APPROACHES FOR MODELLING HOST–PARASITE COEVOLUTION

Theoreticians employ a variety of methods to analyse models of host–parasite coevolution. Models may be investigated analytically, numerically or through simulations, with the feasibility of these methods largely determined by the model assumptions. Results can sometimes be determined precisely or approximately using analytical methods, for example by using linear stability analysis to determine if an equilibrium is stable (Otto & Day, 2007). Additional assumptions may also be required to make models analytically tractable (e.g. slow mutation rates, fixed trait variance). When analytical results cannot be obtained, numerical methods may be used instead to find approximate solutions or to carry out parameter sweeps. Simulations may also be used when the system in question cannot be solved analytically or numerically, or to determine the effects of relaxing certain assumptions (e.g. stochasticity, individual heterogeneity, finite population sizes). For example, simulations are often used to analyse stochastic individual‐based models (Frank, 1996b; Gokhale et al., 2013; Howard & Lively, 1998; Lively & Howard, 1994; Xue & Goldenfeld, 2017).

An illustrative example of the use of different methods can be found in the *adaptive dynamics* framework (also known as evolutionary invasion analysis). Adaptive dynamics is an analytical method for determining long‐term evolutionary trait dynamics (Dieckmann & Law, 1996; Hofbauer & Sigmund, 1998; Metz et al., 1995). The method assumes that mutations have small effects, so that mutants are phenotypically similar to the resident population, and that mutations are rare, so that transient dynamics dissipate before the next mutation occurs. The latter assumption implies that there is a separation of ecological and evolutionary timescales, which greatly simplifies the analysis as one need only consider the invasion fitness (long‐term exponential growth rate) of a rare mutant in a resident population at its *dynamic attractor* (often an equilibrium). The (co)evolutionary dynamics of the system may then be determined analytically or numerically by considering the selection gradient (first derivative of the invasion fitness with respect to the mutant trait) and higher order derivatives (Dieckmann & Law, 1996; Hofbauer & Sigmund, 1998; Metz et al., 1995). Stochastic simulations can be used to relax the assumption of small, rare mutations to verify that the results are robust.

Beyond adaptive dynamics, a wide range of mathematical methods has been developed to determine properties of both population genetic and quantitative genetic frameworks (Fisher, 1930; Lande, 1976; Lion, 2018; Wright, 1931). For example, systems based upon linked genes can be examined analytically (under certain assumptions) using the idea of quasi‐linkage equilibria (QLE) (see Nuismer, 2017, Chapter 6). Assuming loose linkage between genes and weak epistasis, linked genes may be assumed to settle quickly into fixed proportions (Kimura, 1965). This may be used to determine linkage disequilibria (which genes most commonly occur alongside which others) and so give properties of evolutionary outcomes (e.g. Nuismer et al., 2005). Other methods have been developed to explore coevolutionary dynamics over relatively short‐term timescales (Day & Proulx, 2004; Gandon & Day, 2009). Efforts have also been made to develop analytical methods which examine evolving trait variances in eco‐evolutionary models (Sasaki & Dieckmann, 2011), although these methods have yet to be applied in the context of host–parasite coevolution.

## 4 KEY FEATURES OF MODELS OF HOST–PARASITE COEVOLUTION

All models make simplifying assumptions about the real world. It is impossible (and not necessarily beneficial) to capture all the complexity of real systems. When constructing a model, one must therefore determine which features to include and which to exclude while also considering the techniques that will be used to carry out the analysis. For example, the most realistic models would allow all possible traits to evolve but such models would be extremely complicated. Most models of coevolution are limited to a small number of focal traits under selection (usually just two), with all others assumed to be fixed or subject to pleiotropy (and hence determined by the focal traits). Model assumptions may be hypothetical, informed by empirical observations or made for the sake of simplicity. In the context of host–parasite coevolution, the most important biological assumptions concern the underlying genetic structure, trade‐offs and population dynamics of the system. Other key assumptions concern how time is modelled (continuous or discrete), whether the dynamics are deterministic or stochastic and whether the model includes spatial structure. Modelling assumptions can be combined in a variety of ways, resulting in a diverse set of models (see Table S1).

To understand how key features of models affect coevolutionary dynamics, we conducted a literature search (Fig. S1) for papers containing theoretical models of host–parasite coevolution (*n* = 219 papers). We primarily based our literature survey on studies identified in Ashby et al. (2019) (*n* = 183 papers), which searched for theoretical models of coevolution on PubMed published between 2000 and 2017 (see Fig. S1 for full search terms). We then extended our survey manually to include additional papers which were published before 2000 or published after 2017 (*n* = 76). For each paper, we determined the traits under selection, whether the models used quantitative or population genetics, the nature of infection genetics (if applicable) and whether they included population dynamics. The possible outcomes (stable monomorphism/polymorphism, fluctuating selection) of each model were also recorded.

The vast majority (81%) of papers considered in our literature survey examine the coevolution of host resistance and parasite infectivity. The remaining studies consider a wide range of traits, including, but not limited to: disease‐induced mortality (Day & Burns, 2003); within‐host replication rate (Kaitala et al., 1997); rate of superinfection (Castillo‐Chavez & Velasco‐Hernández, 1998); parasite dispersal rate (Gandon, 1998); tendency of the parasite to live freely outside of a host (M’Gonigle & Otto, 2011); parasite susceptibility to medical intervention (Alizon, 2020); host tolerance to infection (Best et al., 2014); recovery rate (van Baalen, 1998; Kada & Lion, 2015); intrinsic mortality of the host due to factors other than the disease (Beck, 1984); relative level of investment in different defence strategies (Iranzo et al., 2015); host birth rate (Gandon et al., 2002); sociality (Bonds et al., 2005; Prado et al., 2009); host migration strategy (Schreiber et al., 2000); mating preference (Ashby, 2020a; Ashby & Boots, 2015; Nuismer et al., 2008); reproductive strategy (Hamilton, 1980; Lythgoe, 2000); recombination rate (Gandon & Otto, 2007; Salathé et al., 2008); and mutation rate (Greenspoon & M’Gonigle, 2013; Haraguchi & Sasaki, 1996; M’Gonigle et al., 2009). As most studies concern resistance–infectivity coevolution, we restrict our discussion of the literature to this topic, focusing our attention on the key features of these models.

### 4.1 Genetic structure

Coevolutionary models must make assumptions about the underlying genetics of host and parasite traits and how they are inherited. Will the model track individual genotypes and allele frequencies or will coevolution be modelled phenotypically? Are hosts and parasites haploid, diploid or polyploid? Is there epistasis or are effects between loci additive? The underlying genetics of a system may be modelled in one of two ways. A *population genetics* approach (used in 145 models in the literature survey) assumes that the traits under selection are determined by a small number of genes with relatively large, potentially epistatic, effects. Population genetic models therefore assume that there are a finite number of host and parasite genotypes which may be haploid, diploid or polyploid. However, even a small number of genotypes can render a model analytically intractable and so population genetic models tend either to neglect population dynamics for simplicity, or to be solved numerically or through simulations. In contrast, a *quantitative genetics* approach (used in 38 models in the literature survey) assumes that the traits under selection are determined by many loci with relatively small, additive effects. Quantitative traits are generally modelled as continuous phenotypes, with analysis focusing on how characteristics of the trait distributions evolve (e.g. mean and variance). By modelling continuous traits and ignoring epistasis, quantitative genetic models can provide analytical insights which complement population genetic models and can often include population dynamics while maintaining tractability (see Appendix S1). Whether a population genetic or a quantitative genetic approach is more appropriate depends entirely on the focus of the model. A population genetic approach is clearly more appropriate when traits are determined by a few major genes, especially when epistasis is involved. Major gene effects have been observed, for instance, in bacteria‐phage systems (Scanlan et al., 2011), viral infections of fruit flies (Cogni et al., 2016) and many other infections of plants and animals (Wilfert & Schmid‐Hempel, 2008). A quantitative genetic approach may be more appropriate if many loci are involved, as has been observed in many cases of plant resistance (Corwin & Kliebenstein, 2017), if the genetic details are unknown or if the focus is on evolution at the phenotypic level.

Direct comparisons between population genetic and quantitative genetic models are rare (Frank, 1993b). There is some evidence that the different approaches may produce qualitatively different results but this may be due to other factors, such as whether models include population dynamics. It is also possible for models to include both population genetics and quantitative genetics, for example by considering a multi‐step infection process (Nuismer & Dybdahl, 2016) or by modelling one species using a continuous trait and the other a discrete trait (Akçay, 2017; Yamamichi & Ellner, 2016).

Epistasis can play an important role in coevolutionary dynamics, as mutations may have little effect on fitness in isolation but together may have a very large effect. For instance, negative epistasis has been shown to result in increased fluctuating selection in some models (Fenton & Brockhurst, 2007) and affects the ability of parasites to adapt to their hosts (Ashby et al., 2014a). Population genetic models must also choose whether hosts and parasites are haploid or diploid, which can have both qualitative and quantitative effects on coevolution. For instance, diploidy may reduce the incidence of cycling (Nuismer, 2006), widen the conditions under which local adaptation is observed (Gandon & Nuismer, 2009) or favour assortative mating (Greenspoon & M’Gonigle, 2014). The precise nature of these effects depends upon the model in question (Mostowy & Engelstädter, 2012). Alternatively, ploidy itself may be allowed to evolve, with theoretical models predicting that parasites generally evolve to be haploid whereas their hosts evolve to be diploid (Nuismer & Otto, 2004). Similar conditions have also been shown to favour the evolution of parasitism (M’Gonigle & Otto, 2011). Discussion of the effects of epistasis and ploidy on host–parasite coevolution can be found in Nuismer (2017) (Chapters 5 and 6).

### 4.2 Infection genetics

**FIGURE 2:**
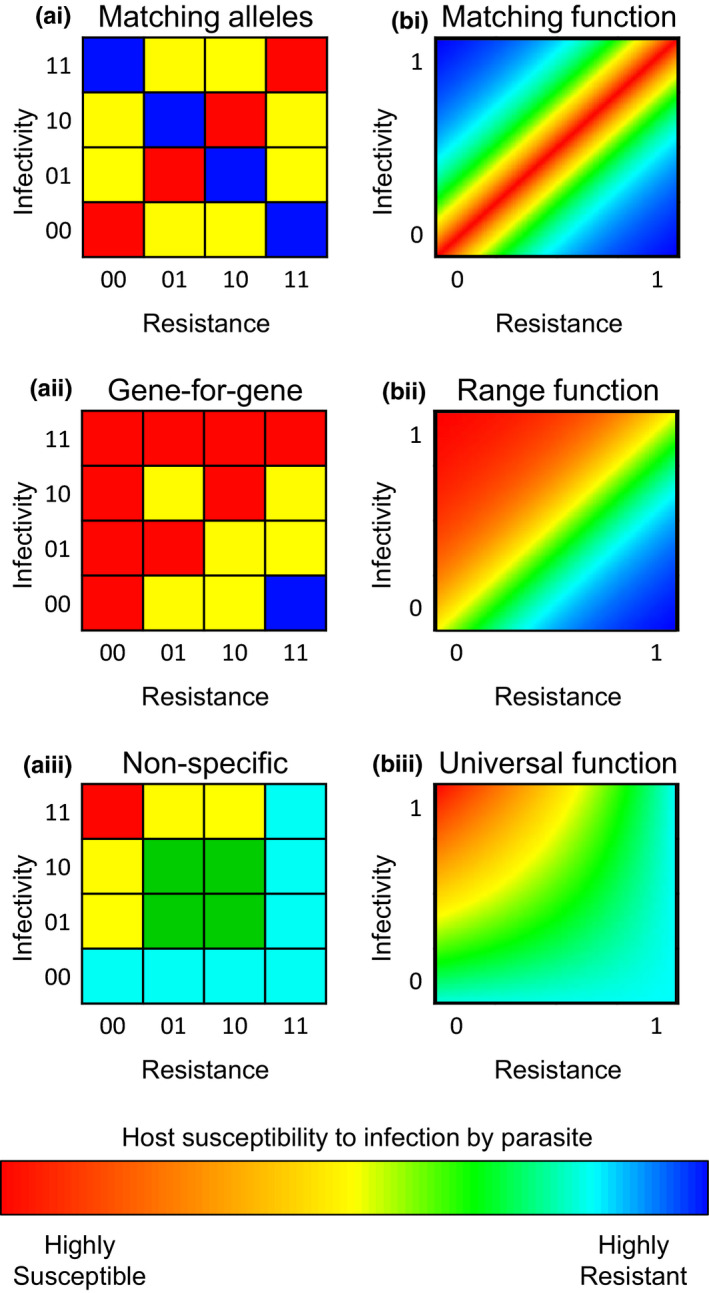
Heatmaps showing example interactions between hosts and parasites, under various models of infection genetics. Red indicates that the host is highly susceptible to the parasite and blue that it is highly resistant. The columns show (a) population genetic and (b) quantitative genetic models. For population genetic models, host and parasite genotypes are indicated by binary strings of length two, with 1 indicating the presence of a resistance or infectivity allele at that locus, giving four possible genotypes in each population. For quantitative genetic models, host and parasite phenotypes are represented by continuous traits between 0 and 1, where 1 indicates the maximum trait value. The rows show example infection genetics when there is (i) high, (ii) varied and (iii) no specificity

The genetic basis of infection and its effects on coevolutionary dynamics are a major focus of the theoretical host–parasite literature. The *infection genetics* of a model describe the interactions between all combinations of hosts and parasites, detailing who can infect whom and to what extent. These generally fall into a few main classes (Table 1; Figure 2). In a population genetic model, there are a finite number of host and parasite genotypes and so the infection genetics can be represented in a table of genotype × genotype interactions, where each host and parasite genotype is defined by a certain set of alleles at one or more genetic loci (Figure 2a). In a quantitative genetic model, the infection genetics are represented by a continuous function of host and parasite traits (Figure 2b). In either case, the infection genetics may vary in the level of specialism or generalism (specificity) with which parasites infect different hosts (and with which hosts can resist different parasites). In most cases, the infection genetics take one of three forms depending on the specificity between host and parasite types: (1) high specificity (Figure 2i); (2) variation in specificity (Figure 2ii) or (3) no specificity (Figure 2iii). A small number of studies have also explored multi‐step infection processes, combining one or more frameworks, but these are relatively rare (Agrawal & Lively, 2003; Fenton et al., 2012; Nuismer & Dybdahl, 2016).

**TABLE 1:** Studies identified in the literature survey with models featuring the different infection genetic systems. Note that some papers compare models with different infection genetics and so may be included multiple times

Genetics	Specificity	Number of studies	Examples
Population genetics	High	98	Hamilton (1980), Seger (1988), Gandon and Otto (2007)
	Varied	69	Frank (1993a), Sasaki (2000), Tellier and Brown (2007a)
	None	4	Forde et al. (2008), Sieber et al. (2014), Frickel et al. (2016)
Quantitative genetics	High	18	Nuismer et al. (2005), Weitz et al. (2005), Williams (2013)
	Varied	16	Sasaki and Godfray (1999), Nuismer et al. (2007), Best et al. (2010)
	None	9	Frank (1994a), Nuismer and Kirkpatrick (2003), Best et al. (2009)

When there is high specificity, either infectivity or resistance is maximized when there is a ‘match’ between the host and parasite traits (Figure 2i). For example, the immune systems of vertebrates are able to detect foreign bodies by means of a self‐nonself recognition system (Medzhitov & Janeway, 2000). Similar ‘matching’ has been observed in bacterial infections of crustaceans (Luijckx et al., 2013), where the parasite must match the host for infection to occur. This is clearly a case of a highly specialized parasite, as it can only infect hosts which it matches (genetically or phenotypically). In a population genetic model, infection occurs if a host and parasite match at some or all loci (Frank, 1991a, 1994b; Gandon et al., 1996; Seger, 1988), which is known as ‘matching alleles’ (MA) (Figure 2ai). Variations exist where the probability of infection depends on the number of matching loci (Nuismer et al., 2017) or where infection occurs if a sufficiently large number of loci match (Shin & MacCarthy, 2016) or if there is a long enough continuous sequence of matching loci (Carrillo‐Bustamante et al., 2015). Conversely, a host may need to ‘match’ the parasite in order to recognize it and mount an immune response, as in the case of the vertebrate major histocompatibility complex (MHC) (Frank, 2002). Such cases are described by ‘inverse matching alleles’ models (Nuismer, 2006). This is also an example of high specificity. Similar infection genetics can be modelled quantitatively, where each host and parasite has a continuous trait value and the probability of infection depends on the similarity between trait values (with infection more likely when they are similar). This is typically referred to as a ‘matching’ or ‘bidirectional’ function (Figure 2bi) and often takes the form of a bell (Gaussian) curve which peaks when host and parasite trait values match (Boots et al., 2014; Nuismer et al., 2005).

When there is variation in specificity, some parasites are able to infect a broader range of hosts than other parasites (conversely, some hosts are able to resist a broader range of parasites than other hosts). As such, there may be variation in the level of generalism among host and parasite types. Such systems have been found to occur in plants (Brown & Tellier, 2011; Flor, 1956), bacteria (Flores et al., 2011; Scanlan et al., 2011), fruit flies (Wilfert & Jiggins, 2010) and crustaceans (Little et al., 2006). In a population genetic framework, a host generally has either a resistant or susceptible allele at each locus, whereas a parasite may or may not possess a corresponding allele at each locus to counter host resistance (note that these are often called ‘virulence’ alleles in the literature, but we refer to these instead as ‘infectivity’ alleles to avoid confusion with disease severity). These are typically referred to as ‘gene‐for‐gene’ (GFG) models (Figure 2aii). In contrast to MA models, GFG models usually assume that the probability of infection is determined by the number of loci at which both the host is resistant and the parasite does not possess a corresponding infectivity allele (Ashby & Boots, 2017; Ashby et al., 2014b; Frank, 1992). In a quantitative genetic framework, variation in specificity is modelled by assuming that the probability of infection is determined by the extent to which an infectivity trait in the parasite exceeds a resistance trait in the host (Best et al., 2010; Nuismer et al., 2007), and is often referred to as a ‘range’ or ‘unidirectional’ model (Figure 2bii).

There has been much debate regarding the relevance of the various infection genetic frameworks to real systems. For example, Parker argued that the GFG system was prevalent in plants (Parker, 1996) whereas Frank believed that their infection genetic systems followed the MA framework (Frank, 1996a, 1996b,b). However, there is evidence that both systems exist across taxa (Dybdahl et al., 2014). Some have argued that the MA and GFG models lie at either end of a spectrum, with the most realistic models somewhere in between (Agrawal & Lively, 2002), but MA models can also be considered as a subset of GFG models (Ashby & Boots, 2017) or GFG models as a subset of inverse matching allele models (Dybdahl et al., 2014). Similarly, comparisons between matching (bidirectional) and range (unidirectional) functions have been studied extensively (Best et al., 2017; Macpherson et al., 2018; Ridenhour & Nuismer, 2007; Yoder & Nuismer, 2010). In general, models that feature fixed, high specificity (i.e. ‘matching’) tend to produce fluctuating selection dynamics more readily. Oscillations are often rapid and may either persist indefinitely (typically as neutrally stable or stochastically driven cycles; Best et al., 2017; Frank, 1991b) or decay towards a stable polymorphic population (damped cycles; Ashby & Gupta, 2014; MacPherson & Otto, 2018). These models typically assume that there are no trade‐offs, so that types only differ in their susceptibility or infectivity profiles. Oscillations are therefore driven by negative frequency‐dependent selection (rare advantage) and occur either between types that have identical levels of specialism or as the result of a ‘chase’ through phenotype space (Best et al., 2017). In contrast, GFG and range (unidirectional) models generally include trade‐offs (see ‘Pleiotropy and trade‐offs’) and types vary in their degree of generalism. These models can therefore produce a far broader range of outcomes, with stable monomorphic or polymorphic populations in either population (Ashby et al., 2019; Cortez et al., 2017; Fenton et al., 2009; Tellier & Brown, 2007a), rapid oscillations occurring within levels of specialism (driven by negative frequency‐dependent selection) or slower oscillations occurring between levels of specialism (driven by trade‐offs) (Ashby & Boots, 2017). Moreover, the oscillations tend to be either stable limit cycles or damped cycles and are not structurally unstable as is common in matching models (Best et al., 2017; Kawecki, 1998; Kwiatkowski et al., 2012). Differences between the outcomes of models with different infection genetic systems, while not surprising, emphasize the need for caution when drawing general conclusions about host–parasite coevolution.

Although most studies explore models with high specificity or variation in specificity, the infection genetics may also be nonspecific. This means that for any two parasite types, A and B, if parasite A is more infective on one host type than parasite B, then it is more infective on all host types (hence, it is said to be ‘universally’ more infective, *sensu* Boots et al., 2014). In a population genetic framework, the universal and GFG models are identical when only one locus is considered but are distinct for multiple loci (Figure 2aiii). For a GFG model, the probability of infection is determined by the number of *corresponding* loci at which the host is resistant and the parasite is not infective, whereas in a nonspecific model, it is the *overall proportion* of resistance and infectivity alleles that determines the probability of infection. In a quantitative genetic framework, the difference between universal and range models is more subtle. Range models are typically close to step functions, with parasites having very high infectivity on a subset of hosts and very low infectivity on all others (Figure 2bii), whereas universal models generally incorporate a broader spectrum of levels of infectivity, with different parasites able to infect different hosts to varying degrees (Figure 2biii).

### 4.3 Pleiotropy and trade‐offs

**FIGURE 3:**
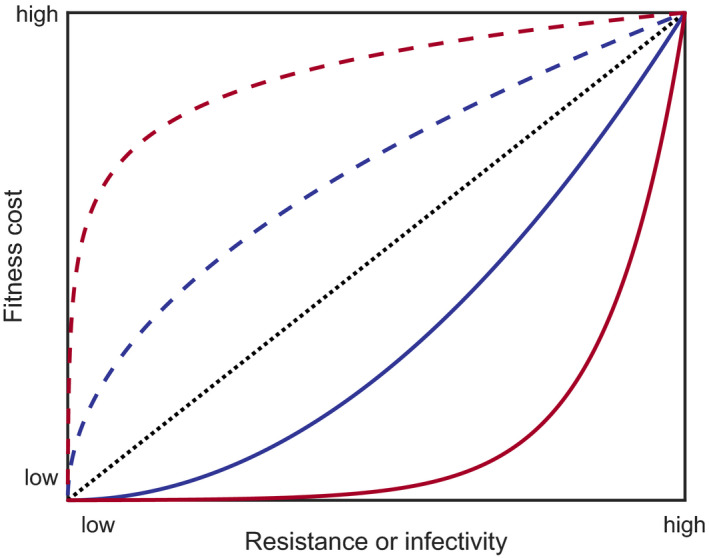
Examples of different trade‐off shapes. Trade‐offs may be linear (dotted), accelerating (solid) or decelerating (dashed). Non‐linear trade‐offs may be strongly (red) or weakly (blue) accelerating or decelerating as resistance or infectivity increases

Coevolutionary models often incorporate pleiotropy through trade‐offs between life‐history traits. This reflects empirical observations where, for example, increased host resistance may be associated with decreased growth or reproduction (Bartlett et al., 2018; Wright et al., 2016) or a higher within‐host replication rate may lead to greater parasite transmissibility but may also be associated with higher disease‐induced mortality (and hence, a shorter infectious period) (de Roode et al., 2008; Thrall & Burdon, 2003). Such trade‐offs are usually not necessary in models with high specificity because selection may be driven by negative frequency dependence. In contrast, if there were no trade‐offs associated with resistance or infectivity in models with variable or no specificity, then the most resistant host and the most infective parasite would always have the highest fitness. Trade‐offs can be incorporated into models by letting other life‐history parameters vary as functions of a focal trait (Figure 3). The precise trade‐off will depend on the system being modelled and may be hypothetical or estimated from empirical results (Jessup & Bohannan, 2008). Where empirical data are not available, the effects of varying the shape and magnitude of any trade‐offs should be considered in the model analysis, as these can both quantitatively and qualitatively affect the results.

It is well known that the shape and strength of trade‐offs can have significant effects on evolutionary dynamics (Bowers et al., 2005; Kisdi, 2006). For example, changing the strength of fitness costs can cause the dynamics to switch between stable equilibria and fluctuating selection in models of host–parasite coevolution (Best et al., 2010; Fenton & Brockhurst, 2007; Sasaki, 2000). Trade‐off shapes (Figure 3) can also cause qualitative shifts in coevolutionary dynamics, with stable monomorphism more likely when there are diminishing fitness returns (e.g. costs accelerate; Kisdi, 2006) and evolutionary branching (leading to stable polymorphism) more likely when trade‐offs are close to linear or are slightly decelerating (Bowers et al., 2005).

### 4.4 Population dynamics

**FIGURE 4:**
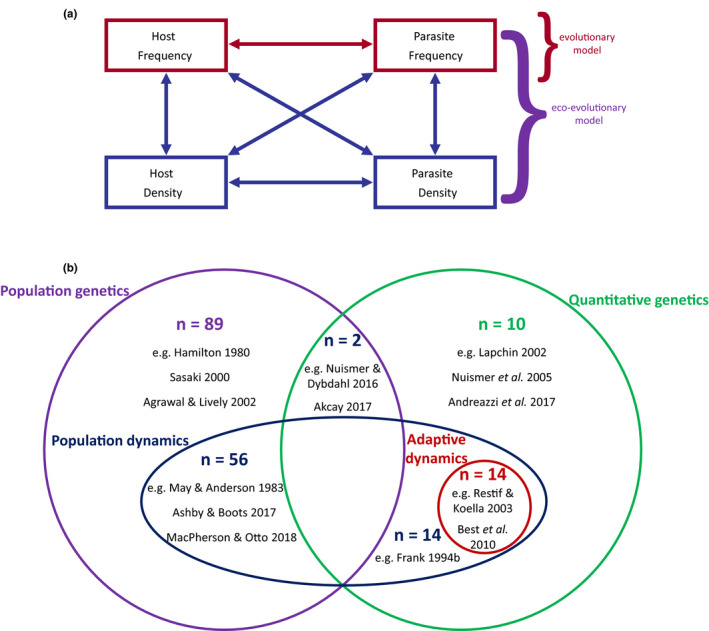
Population dynamics can be included or excluded from models of host–parasite coevolution. (a) Models which do not include population dynamics only consider frequency‐dependent dynamics (red) and hence excluding population dynamic results in the loss of many potentially important density‐dependent effects (blue). (b) Broad classification of models in the literature survey, showing the number of models of each type along with examples (see Appendix S1 for more details)

Population dynamics (also referred to as *ecological or epidemiological dynamics*) describe changes in the number (or density) of individuals in a population over time. Models of host–parasite coevolution can be broadly divided into two categories based on whether they include (84 models in the literature survey) or exclude (101 models in the literature survey) population dynamics (Figure 4b). Many models neglect population dynamics by assuming either that they do not influence fitness, or that the host and parasite population sizes are constant or infinite (Fenton et al., 2009; Mostowy & Engelstädter, 2012; Sasaki, 2000). In the absence of population dynamics, selection is only frequency‐dependent, as fitness depends on the relative proportions of individuals with different genotypes/phenotypes and not on their absolute abundance (Ashby et al., 2019; MacPherson et al., 2021a). Population dynamics are often neglected for simplicity, especially to allow for more detailed genetic effects (e.g. epistasis, ploidy) without the model becoming analytically intractable. In contrast, *eco‐evolutionary models* assume that population dynamics are integral to (co)evolution, as the evolution of a trait will generally affect, and be affected by, the population dynamics of hosts and parasites. Selection is therefore both frequency‐ and density‐dependent, with feedback loops existing between population dynamics and evolutionary dynamics (Ashby et al., 2019; Figure 4a). However, eco‐evolutionary feedback loops can limit analytical tractability and so it is often necessary to make simplifying assumptions about the genetics or mutations (Dieckmann & Law, 1996; Metz et al., 1995), or use numerical or simulation‐based approaches.

Population dynamics were first incorporated into coevolutionary models by Pimentel (1961), who also suggested that their effects on coevolution might be significant in an empirical context (Pimentel et al., 1978). Several early studies of host–parasite coevolution also emphasized the importance of eco‐evolutionary feedbacks (Frank, 1991a; May & Anderson, 1983). In some cases, population dynamics have little or no impact on coevolution (Nuismer, 2017). However, it is well established that population dynamics often have significant qualitative and quantitative effects on host–parasite coevolution models due to eco‐evolutionary feedbacks (Frank, 1991a). A number of recent studies have directly explored the effects of introducing eco‐evolutionary feedbacks into (or removing them from) models of host–parasite coevolution, leading to fundamental changes in model outcomes (Ashby et al., 2019; Gokhale et al., 2013; MacPherson & Otto, 2018). To see why eco‐evolutionary feedbacks can be so important, consider selection for an allele that confers resistance to a common parasite. As the allele increases in frequency, fewer parasites will be able to infect hosts and so there is likely to be a reduction in parasite prevalence, and hence weaker selection for resistance. At the same time, the strength of selection for counter‐adaptations in the parasite will tend to increase, although there may be a reduction in the mutation supply (due to the smaller parasite population). In a model that lacks population dynamics, there is no change in the parasite population size and so there is no impact on the strength of selection in the host, nor is there any impact on mutation supply.

Our survey of the literature suggests that models which include population dynamics are more likely to lead to stable polymorphism (see Appendix S1). Indeed, in some models, population dynamics have been shown to be necessary for stable polymorphism (Ashby et al., 2019). Theoretical studies have also shown that population dynamics tend to dampen oscillations in allele frequencies or make fluctuating selection less likely (Ashby et al., 2019; MacPherson et al., 2021b; MacPherson & Otto, 2018). Population dynamics can also cause a quantitative shift in evolutionary outcomes, although the precise effects depend on the model.

### 4.5 Time

An important, but rarely discussed, aspect of many models is whether time proceeds continuously (76 models in the literature survey) or in discrete steps (108 models in the literature survey). When modelling a specific biological system, there may be strong motivation to take one approach over the other, but in a general model the choice of how to represent time may be made arbitrarily or for convenience. The question of how to model time is often considered in the context of generational overlap: some models assume that generations of hosts (and sometimes also parasites) are separate, whereas others allow them to overlap. If generations do not overlap, then the entire population must be replaced at each time step.

When time is modelled in discrete steps, generations may or may not overlap. In the case of non‐overlapping generations, each generation of hosts is born at the same time, with infection, reproduction and death occurring in each time interval. This is a reasonable approximation for many biological systems, including certain annual plants (Austerlitz et al., 2000) and insects (Bjørnstad et al., 2016) for which generations do not overlap. Alternatively, discrete time may be used when generations do overlap, but when there are strong, periodic effects relating to infection, reproduction or death. For instance, seasonal effects may cause all births, infections and deaths to occur in specific seasons. In either case, a similar modelling approach is used. In a deterministic setting, the population at time *t* + 1 is entirely determined by the population at time *t*. These models are formulated as recurrence relation‐style difference equations, where the frequencies (and potentially densities) of hosts and parasites of each type at a given time step are functions of the populations at the previous time step. For example, in a population with *n* host types and *m* parasite types, the frequency dynamics are given by equations of the form:

(1) $$h_{t+1}^{i}=f_{i}\left(h_{t}^{1},h_{t}^{2},\cdots ,h_{t}^{n},p_{t}^{1},p_{t}^{2},\cdots ,p_{t}^{m}\right)$$

(2) $$p_{t+1}^{i}=g_{i}\left(h_{t}^{1},h_{t}^{2},\cdots ,h_{t}^{n},p_{t}^{1},p_{t}^{2},\cdots ,p_{t}^{m}\right)$$

where *f_i_* and *g_i_* are functions that describe the change in host and parasite types *i*, and $h_{t}^{i}$ and $p_{t}^{i}$ are the frequencies of the *i*th host and parasite types respectively at time *t*. In a stochastic setting, the population at time *t *+ 1 depends probabilistically on the population at time *t*, and so one would use a synchronous simulation algorithm to update the populations to account for random events.

For organisms with life cycles in which generations overlap and where processes such as infection and reproduction can occur at any time, continuous time models are more realistic. Continuous time models may be formulated through sets of ordinary or partial differential equations, for example:

(3) $$\frac{dh_{i}\left(t\right)}{dt}=F_{i}\left(h_{1}\left(t\right),h_{2}\left(t\right),\cdots ,h_{n}\left(t\right),p_{1}\left(t\right),p_{2}\left(t\right),\cdots ,p_{m}\left(t\right)\right)$$

(4) $$\frac{dp_{i}\left(t\right)}{dt}=G_{i}\left(h_{1}\left(t\right),h_{2}\left(t\right),\cdots ,h_{n}\left(t\right),p_{1}\left(t\right),p_{2}\left(t\right),\cdots ,p_{m}\left(t\right)\right)$$

where *F_i_* and *G_i_* are functions which describe the change in host and parasite types *i*, and *h_i_* (t) and *p_i_* (*t*) are the frequencies of the *i*th host and parasite types respectively at time *t*. Stochastic versions of a continuous time model usually use the Gillespie (asynchronous) stochastic simulation algorithm (Gillespie, 1976).

**FIGURE 5:**
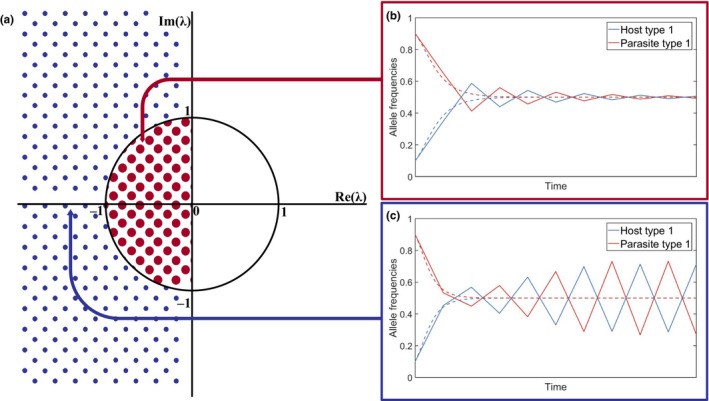
A comparison of continuous (dashed) and discrete (solid) time models. (a) Different regions of the complex plane, defined by the real (Re(*λ*)) and imaginary (Im(*λ*)) parts of eigenvalues, *λ*, display different dynamics. (b) A stable equilibrium for both discrete and continuous time models (red region). (c) A stable equilibrium for continuous time models and unstable cycles for discrete time models (blue region). The models used to generate these dynamics are described in Appendix S2

The use of discrete or continuous time can have significant effects on model dynamics (May, 1973a). Discrete time models have a greater tendency to ‘overshoot’ an equilibrium because time proceeds in fixed jumps, whereas continuous time models may approach an equilibrium smoothly. Mathematically, the stability of an equilibrium is determined by the *eigenvalues* of a linear approximation to the system (for an introduction to eigenvalues, see Otto & Day, 2007). In a discrete time model, an equilibrium is only stable if all eigenvalues have absolute value less than 1. All eigenvalues must therefore lie within a circle of radius 1, centred at the origin, in the complex plane (Figure 5a). In a continuous time model, stability only requires the real part of all eigenvalues to be negative. All eigenvalues must therefore lie to the left of the imaginary axis (Figure 5a). Hence, whether a model is implemented in discrete time or continuous time can greatly affect the stability of an equilibrium. For example, Kouyos et al. (2007) showed that adapting a discrete time model of host–parasite coevolution to continuous time causes cycles in allele frequencies to become damped. Given that cycling is of special interest in models of host–parasite coevolution (in part due to links with the Red Queen Hypothesis for sex (Lively, 2010), although cycling is not necessary for the maintenance of sex (Ashby, 2020b)), one must be careful to ensure that oscillations are not simply artefacts that arise due to discrete time. Conversely, if a biological system is better approximated by a discrete time model, then a continuous time model may underestimate the potential for cycles.

### 4.6 Stochasticity

**FIGURE 6:**
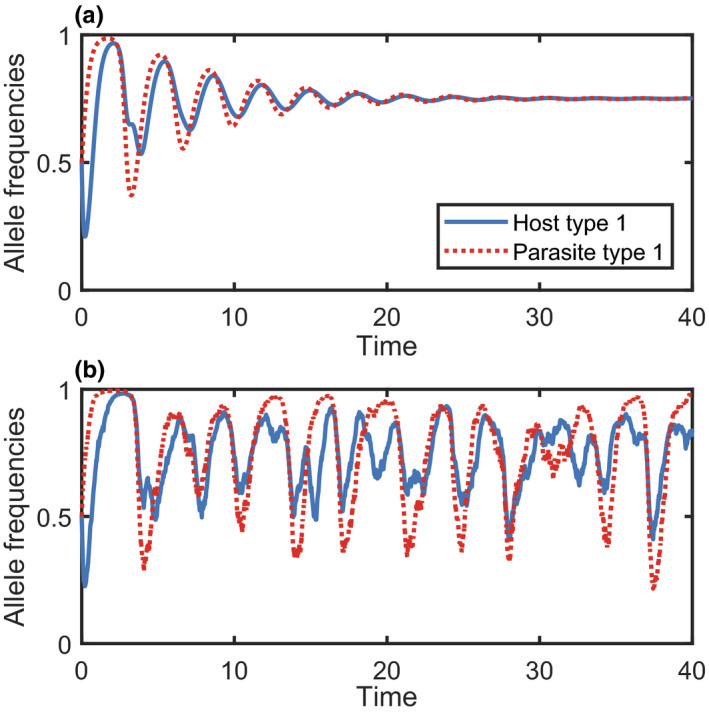
Example time series for allele frequencies in a single locus model with matching allele infection genetics, implemented (a) deterministically and (b) stochastically. The deterministic model tends towards a stable equilibrium, whereas the stochastic model is continually pushed away from the equilibrium, leading to noise‐induced oscillations. The model used to generate these dynamics is described in Appendix S3

In nature, there is an element of chance to all processes. Whether or not an individual reproduces or is infected at a particular moment in time will depend on a variety of factors, many of which will be subject to randomness (e.g. finding a mate, encountering a parasite). Deterministic models (97 models in the literature survey; Figure 6a), such as those in (1), (2), (3), (4), assume that stochasticity is relatively unimportant, thus greatly simplifying the analysis. This is often a reasonable approximation in large populations, as the effects of demographic stochasticity decrease with the square root of population size (May, 1973b). Stochasticity (88 models in the literature survey; Figure 6b) may be incorporated using stochastic differential equations, which include noise terms, or through simulations (for instance by allowing infection to occur with a certain probability). However, stochastic models are computationally more intensive than their deterministic counterparts, with computational time increasing rapidly with population size. Moreover, many replicates may be required to reveal representative dynamics. Hybrid models which combine deterministic and stochastic methods may be computationally more efficient when modelling processes that occur at contrasting spatial or temporal scales. For example, one may choose to model short‐term population dynamics deterministically, but model rare mutations stochastically (Ashby et al., 2019; Best et al., 2010).

Incorporating stochasticity can have significant effects on coevolutionary dynamics. Crucially, stochasticity can cause rare types to go extinct, potentially causing systems with negative frequency‐dependent selection to go to fixation rather than cycle indefinitely (Gokhale et al., 2013). However, stochasticity can also induce cycling by repeatedly pushing a system away from a stable equilibrium (Figure 6; Kouyos et al., 2007; Lythgoe, 2000; M’Gonigle et al., 2009). Stochasticity may also prevent stable polymorphism by causing sub‐populations to go extinct (Schenk et al., 2018; Xue & Goldenfeld, 2017).

### 4.7 Spatial structure

**FIGURE 7:**
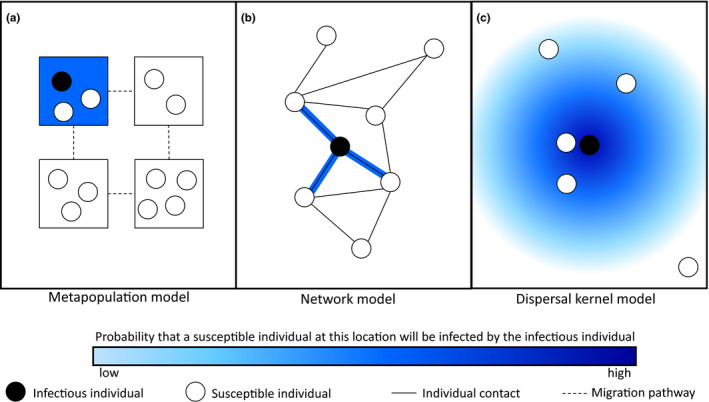
Different ways of modelling spatial structure. (a) In a metapopulation model, the population is divided into distinct sub‐populations (or patches) with infection occurring through random mixing within each subpopulation and migration between patches. (b) In a network model, an individual can only infect its social or sexual contacts, as indicated by edges between nodes (individuals). (c) In a continuous space model, the probability of infection decreases with distance using a *dispersal kernel*

Models of host–parasite coevolution often assume that the populations are well‐mixed (as in 158 models in the literature survey) so that all individuals have an equal probability of encountering or interacting with all others. By using the law of mass action and replacing many individual interactions with an average over the population (known as a *mean‐field approximation*), well‐mixed models can reduce a complex many‐body problem to a relatively simple one‐body problem. In other words, one can approximate the transmission dynamics of a large number of randomly mixing infectious and susceptible hosts by a single term, *βSI*, where *β* is the transmission rate and *S* and *I* are the densities of susceptible and infectious hosts. Although this approach often works well, individuals may instead interact strongly with a small subset of the population due to social or sexual contact networks, or due to proximity arising from spatial structure. Models may incorporate spatial structure (27 models in the literature survey) in a number of different ways, including metapopulations (Figure 7a), where migration occurs between distinct, well‐mixed patches (Frank, 1991; Gandon et al., 1996; Gomulkiewicz et al., 2000; Nuismer et al., 1999; Lively, 1999), individual‐based models on lattices or networks (Figure 7b), where the contact structure of the population is represented by a collection of edges (contacts) between nodes (individuals; Ashby et al., 2014b; Lion & Gandon, 2015) and models with continuous space (Figure 7c) where the probability of infection decreases with distance (known as a dispersal kernel).

Spatial structure has been shown to have several important effects on host–parasite coevolution (Lion & Gandon, 2015). For example, spatial structure tends to promote fluctuating selection (Gómez et al., 2015), greater host resistance (Ashby et al., 2014b) and lower infectivity (Best et al., 2011). Spatial structure has also been shown to favour the evolution of more nested infection genetic systems (Valverde et al., 2017). Models predict that greater environmental heterogeneity promotes generalism in hosts and parasites (Hesse et al., 2015) and increases both global and local polymorphism (Frank, 1991b; Tellier & Brown, 2011). Unlike well‐mixed models, spatially structured models allow for local adaptation, especially in metapopulations (Nuismer, 2006; Thrall et al., 2016). For example, parasites are predicted to be most highly locally adapted when they have high migration rates relative to their hosts (Gandon et al., 1996; Morgan et al., 2005). Local maladaptation can also occur (see Chapter 10 of Nuismer (2017)) and appears to be more common in population genetic models than in quantitative genetic models (Ridenhour & Nuismer, 2007).

## 5 RECENT DEVELOPMENTS & FUTURE DIRECTIONS

Historically, models of host–parasite coevolution have tended to focus on a few key areas of interest, in particular: (1) the role of genetics and specificity in determining coevolutionary dynamics; (2) the evolution and maintenance of sex and recombination (the Red Queen Hypothesis); and (3) spatial and temporal patterns of adaptation and diversity. A common theme through much of this body of work has been the focus on relatively simple systems, usually pairwise models of a single host species and a single parasite species with straightforward infection genetics. This is not a criticism; it is entirely reasonable (and prudent) to understand the dynamics of simple systems before considering more complex scenarios. Recently, however, there has been a noticeable trend towards increasing complexity in models of host–parasite coevolution, whether in the form of hybrid models to understand the role of eco‐evolutionary feedbacks (Ashby et al., 2019; Gokhale et al., 2013; MacPherson & Otto, 2018), more complex models of infection and defence (Akçay, 2017; Iranzo et al., 2015; Nuismer & Dybdahl, 2016; Weinberger et al., 2012) or additional species interactions (Best, 2018; King & Bonsall, 2017; Kwiatkowski et al., 2012; Seppälä et al., 2020). Here, we summarize some of the recent developments in the literature and discuss likely directions for future theoretical research.

There has been a noticeable effort in recent years to bridge the gap between models that include and exclude population dynamics and hence to understand how eco‐evolutionary feedbacks affect host–parasite coevolution (Ashby et al., 2019; Gokhale et al., 2013; MacPherson & Otto, 2018; Schenk et al., 2020). Although the importance of population dynamics in models of host–parasite coevolution was first highlighted by May and Anderson (1983), these dynamics are still routinely overlooked in theoretical studies of coevolution. As a result, two largely independent bodies of host–parasite coevolutionary theory exist based on the inclusion or exclusion of population dynamics. Given that we know eco‐evolutionary feedbacks can fundamentally change coevolutionary outcomes in different types of models, a key challenge for future research is to determine to what extent our overall theoretical understanding of host–parasite coevolution depends on the absence of population dynamics. This is not necessarily straightforward, as models with and without population dynamics are rarely directly comparable, but methods have been developed to introduce eco‐evolutionary feedbacks into models that lack population dynamics (Ashby et al., 2019).

A second major area of recent theoretical research is the exploration of more complex systems of infection and defence, often motivated by specific biological mechanisms, including human leukocyte antigens (Penman & Gupta, 2017); vertebrate adaptive immunity (Lighten et al., 2017; Nourmohammad et al., 2016); prophage sequences (Nadeem & Wahl, 2017) and CRISPR‐Cas systems in bacteria (Childs et al., 2012, 2014); constitutive and induced defences (Kamiya et al., 2016); and multi‐step infection processes (Nuismer & Dybdahl, 2016; Shin & MacCarthy, 2016). Sequencing is now relatively cheap and widely accessible compared to when the first theoretical frameworks for infection genetics were developed, yet it remains unclear to what extent the classical models of infection genetics (MA and GFG) are representative of real biological systems beyond a few specific examples (e.g. Luijckx et al., 2013; Thrall & Burdon, 2003). New genomic techniques are also likely to identify more realistic infection genetics that do not fit neatly into current frameworks (Ebert & Fields, 2020). For many biological systems, it is still unknown whether resistance and infectivity are caused by a few major loci (Wilfert & Schmid‐Hempel, 2008) or many loci with smaller effects (Corwin & Kliebenstein, 2017); the relevance of population and quantitative genetic approaches to many systems is therefore uncertain. More empirical data on the nature of the underlying genetics would greatly help to inform future theoretical models. Similarly, real‐world trade‐offs also tend to be poorly characterized. For instance, the relationship between transmission, virulence and recovery rate is still not well understood (Acevedo et al., 2019), even though such trade‐offs are commonly modelled. Furthermore, the shape of trade‐offs (accelerating or decelerating) is often difficult to detect empirically, and therefore, most studies are only able to identify that a trade‐off exists rather than reveal precise details about its shape (Bartlett et al., 2018). A key strength of theoretical models is the ability to vary parameters and trade‐offs at will, but a better understanding of real‐world trade‐offs would help to inform which theoretical trade‐offs are most relevant or common. Better integration between theoretical models and empirical data will be a key direction for future research, especially with respect to infection genetics and trade‐offs.

A third significant, albeit rather limited, area of recent research has been the development of coevolutionary models that include additional species interactions (Best, 2018; King & Bonsall, 2017; Kwiatkowski et al., 2012; Seppälä et al., 2020), mirroring a shift in experimental evolution research away from pairwise interactions (Buckling & Rainey, 2002) to more complex communities (Castledine et al., 2020; Friman & Buckling, 2013; Hall et al., 2020; Nuismer & Doebeli, 2004; Rafaluk‐Mohr et al., 2018). The limited theoretical work in this area thus far has explored host–parasite coevolution in the presence of a predator (Best, 2018), multiple parasites (Mostowy et al., 2010; Seppälä et al., 2020) or defensive symbionts (King & Bonsall, 2017; Kwiatkowski et al., 2012). A crucial challenge for future theoretical research is not only to explore a much wider range of ecological interactions (along with more varied genetic and environmental assumptions), but also to predict how species interactions more generally mediate host–parasite coevolution. There are infinitely many communities that one could model, but can we make general predictions about how certain types of species interactions affect host–parasite coevolution, based on factors such as trophic level and where they lie on the antagonistic‐mutualistic continuum? Furthermore, do the specific interactions in larger communities matter, or can we make general predictions about the nature of host–parasite coevolution based on the number and type of interactions within the community?

Future theoretical research will also likely consider greater complexity in two further areas: host heterogeneity and multi‐scale models. For example, despite empirical evidence that host life‐history traits vary significantly with age (Bruns et al., 2017; Lian et al., 2020) and theoretical predictions that age‐structure affects disease dynamics (Clark et al., 2017), host heterogeneity in the form of age‐specific resistance has only focused on single trait adaptation (Ashby & Bruns, 2018) and has yet to be explored in the context of host–parasite coevolution. Similarly, multi‐scale models, which capture both within‐ and between‐host dynamics, have largely considered parasite evolution, with few studies considering the impact of selection across scales on host–parasite coevolution (Gilchrist & Sasakiz, 2002; Pugliese, 2011).

## 6 CONCLUSION

Theoretical models play a key role in our understanding of host–parasite coevolution. These models encompass a wide variety of biological assumptions and technical approaches, making it difficult to reconcile contrasting predictions and to determine how different factors influence coevolutionary outcomes. Our literature survey revealed that population dynamics and the underlying infection genetics of the system are consistently key factors in determining coevolutionary dynamics, although less‐considered factors such as stochasticity and how time is modelled can also significantly affect model outcomes (see Box 1). Recent research has largely focused upon understanding the effects of more realistic ecological and genetic assumptions on host–parasite coevolution, but the effects of host heterogeneity and selection across scales have received little attention. We anticipate future theoretical research will continue to incorporate more realistic assumptions about host–parasite life‐history traits and the wider environment but care must be taken that increased complexity is empirically motivated and is not pursued purely for complexity's sake.

## CONFLICT OF INTEREST

The authors have no conflict of interest to declare.

### PEER REVIEW

The peer review history for this article is available at https://publons.com/publon/10.1111/jeb.13981.

## Supporting information

Supplementary Material

## Data Availability

The data that supports the findings of this study are available in the supplementary material of this article. They have also been uploaded to Dryad (DOI: doi.org/10.5061/dryad.2ngf1vhq2).
