# Supplementary material for: Coevolutionary theory of hosts and parasites
Source: J Evol Biol. 2022 Jan 30;35(2):205–24. doi: 10.1111/jeb.13981 (PMC9305583; doi:10.1111/jeb.13981)
Supplement: Supplementary file 1 — Supplementary Material [file JEB-35-205-s001.zip › jeb13981-sup-0001-FigS1.pdf]

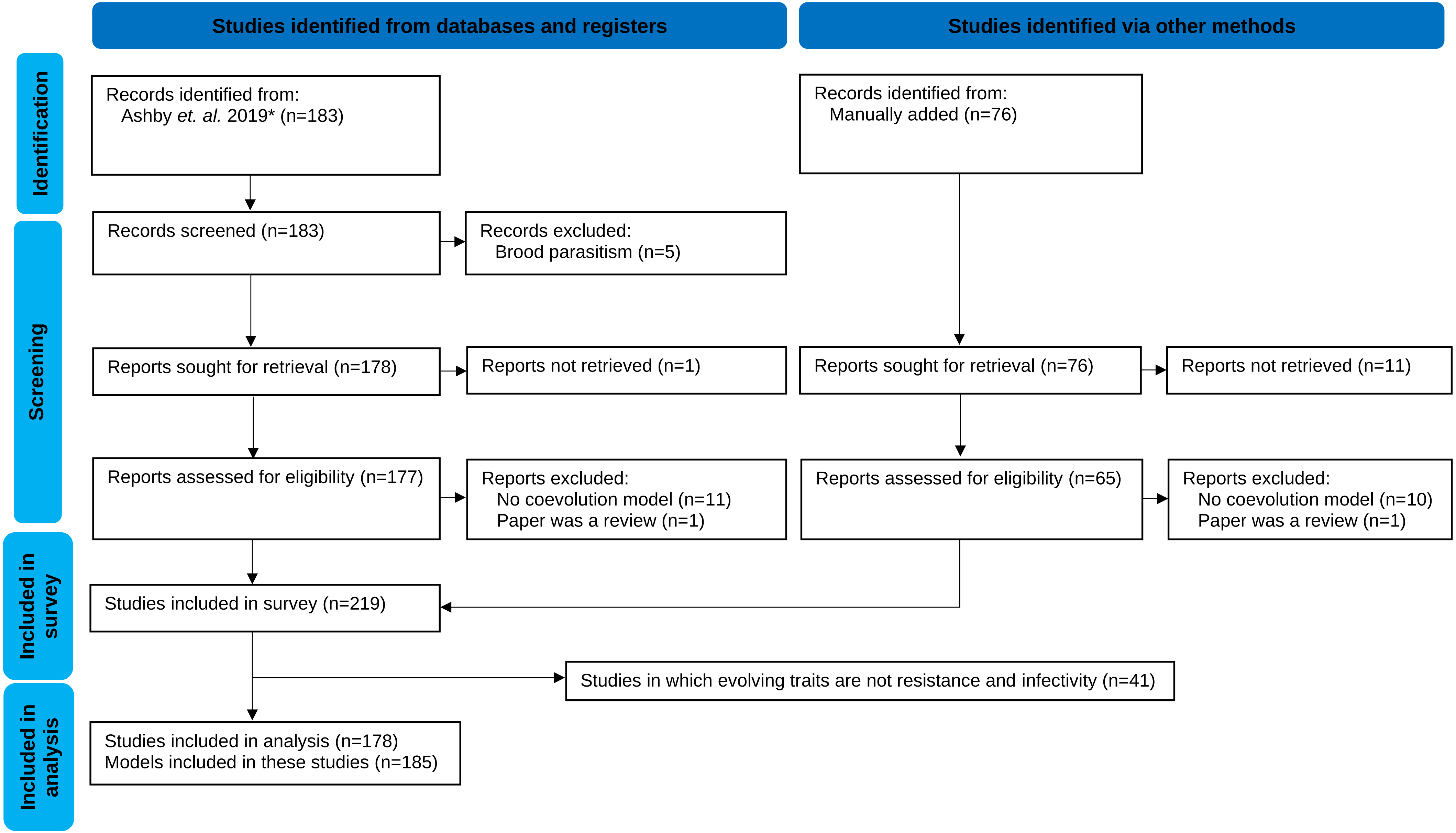

* Pubmed search for "(model* OR simulation* OR theory OR theoretical OR mathematical) AND (coevolution* OR co-evolution* OR coevolve* OR co-evolve* OR (red AND queen)) AND (host* OR parasite* OR pathogen*) AND ("2000"[Date - Publication] : "2017"[Date - Publication])".
