# Supplementary material for: Coevolutionary theory of hosts and parasites
Source: J Evol Biol. 2022 Jan 30;35(2):205–24. doi: 10.1111/jeb.13981 (PMC9305583; doi:10.1111/jeb.13981)
Supplement: Supplementary file 1 — Supplementary Material [file JEB-35-205-s001.zip › jeb13981-sup-0002-TableS1.pdf]

	Population Dynamics	Population Genetics	Quantitative Genetics	High Specificity	Varied Specificity	No Specificity	Spatial Structure	Stochasticity	Continuous Time	Discrete Time
Population Dynamics	84	56	28	51	33	12	14	41	58	27
Population Genetics		147	2	99	70	4	21	72	54	95
Quantitative Genetics			40	19	17	9	6	18	23	17
High Specificity				117	39	3	20	57	44	75
Varied Specificity					86	3	12	36	28	58
No Specificity						13	3	6	11	2
Spatial Structure							27	11	8	19
Stochasticity								88	40	49
Continuous Time									77	2
Discrete Time										110

Table S1: The different combinations of assumptions made in the models from the literature survey. There are 185 models in total. Note that some models are considered in the context of several different systems of infection genetics (hence the specificity categories sum to more than 185).
