# Supplementary material for: Coevolutionary theory of hosts and parasites
Source: J Evol Biol. 2022 Jan 30;35(2):205–24. doi: 10.1111/jeb.13981 (PMC9305583; doi:10.1111/jeb.13981)
Supplement: Supplementary file 1 — Supplementary Material [file JEB-35-205-s001.zip › jeb13981-sup-0003-AppendixS1.pdf]

Coevolutionary Theory of Hosts and Parasites

Appendices

Lydia J. Buckingham[1,2] and Ben Ashby[1,2]

[1] Department of Mathematical Sciences, University of Bath, Bath. UK. BA2 7AY
[2] Milner Centre for Evolution, University of Bath, Bath. UK. BA2 7AY

1 Literature Survey

We undertook a survey of host-parasite co-evolution models. We identified 185 models which explicitly explored the co-evolution of host resistance and parasite infectivity (Fig. S1). These models were categorised depending upon whether or not they incorporated population dynamics, whether they used quantitative or population genetics, and whether or not they generated stable, trait polymorphism in their results. Certain key associations were identified between these categories, which are described below.

Firstly, we grouped studies according to their genetic structure and whether they included or excluded population dynamics (Table S1). Note that two of the models in the literature survey used multi-step infection genetics which included both population genetics and quantitative genetics elements; these are not included in our analysis.

	Pop. Gen.	Quant. Gen.	Total
Pop. Dynamics	56	28	84
No Pop. Dynamics	89	10	99
Total	145	38	183

Table S1: Summary of theoretical studies categorised by genetics and population dynamics.

We can see that 74% of quantitative genetics models included population dynamics whereas only 39% of population genetics models included population dynamics. This suggests that quantitative genetics models may be more likely than population genetics models to include population dynamics.

Secondly, we grouped studies according to whether they exhibited stable polymorphism or not and whether they included or excluded population dynamics (Table S2). Note that twelve of the models in the literature survey did not include details of whether or not they could generate stable, trait polymorphism; these are not included in our analysis.

	Polymorphism	No Polymorphism	Total
Pop. Dynamics	67	12	79
No Pop. Dynamics	64	30	94
Total	131	42	173

Table S2: Summary of theoretical studies categorised by population dynamics and polymorphism.

We can see that 85% of population dynamics models generated polymorphism whereas only 68% of models without population dynamics generated polymorphism. This suggests that models which include population dynamics may be more likely to lead to polymorphism than models which exclude population dynamics.

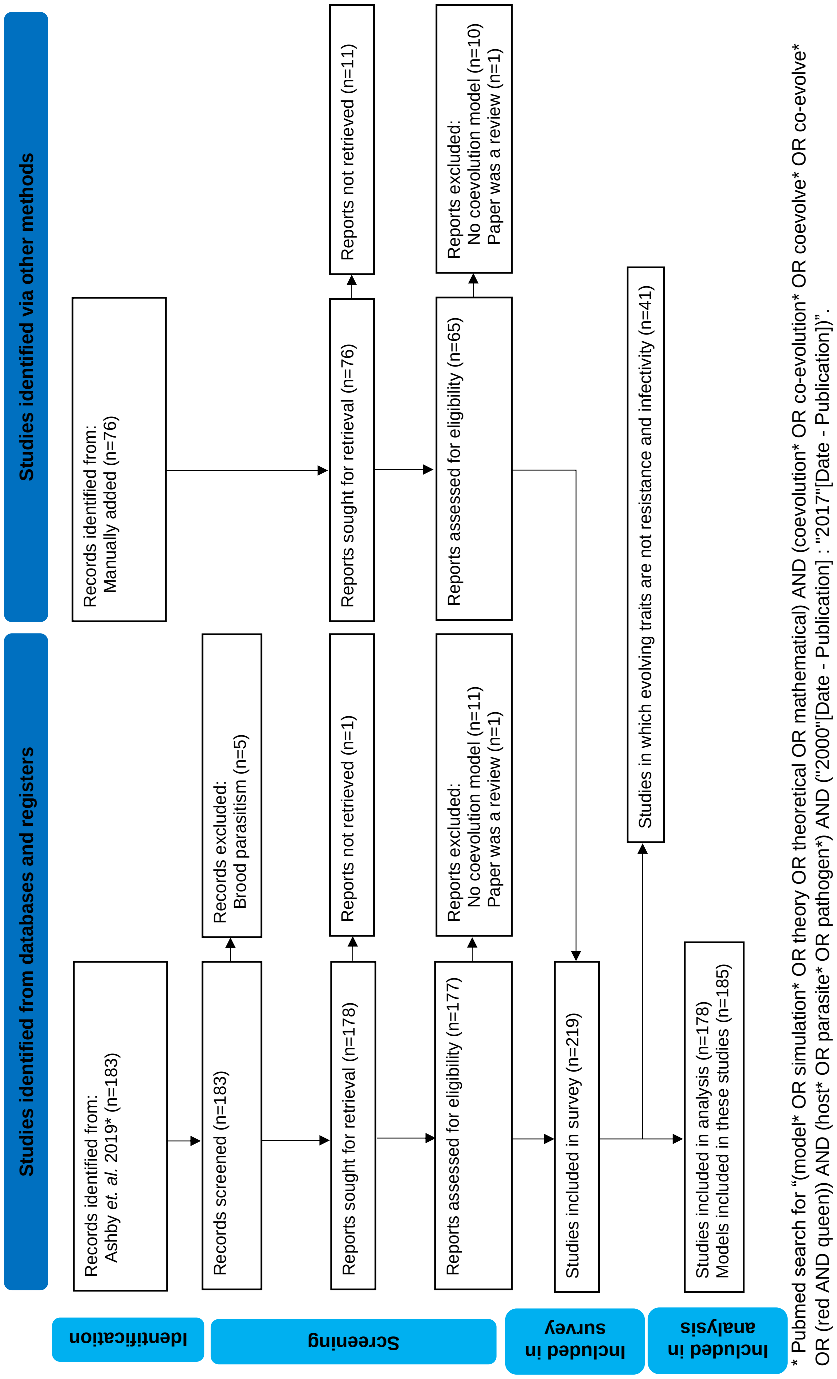

Figure S1: PRISMA diagram describing the process of selecting papers for inclusion in the literature survey.

2 Discrete/Continuous Time Model

Figure 5 in the main text looks at the effects of using discrete versus continuous time. The time series shown in this figure are generated using the following model. Consider two sub-populations of hosts interacting with two sub-populations of parasites, where h_i is the frequency of hosts of type i and p_i is the frequency of parasites of type i, for $i \in \{1, 2\}$. Note that $h_1 + h_2 = 1$ and $p_1 + p_2 = 1$. We model this situation using the following system, formulated in terms of discrete and continuous time:

	Discrete	Continuous	
(1)	$h_1^{t+1} = h_1^t(1 + f_1^t - \bar{f}^t)$	$\frac{\mathrm{d}h_1}{\mathrm{d}t} = h_1(f_1 - \bar{f})$	(2)
(3)	$p_1^{t+1} = p_1^t(1 + g_1^t - \bar{g}^t)$	$\frac{\mathrm{d}p_1}{\mathrm{d}t} = p_1(g_1 - \bar{g})$	(4)

Here, h_i^t represents the value of h_i at time t, f_i is the fitness of host type i and $\bar{f}$ is the average fitness of hosts, given by:

$$\bar{f} = f_1 h_1 + f_2(1 - h_1) \tag{5}$$

$$\bar{g} = g_1 p_1 + g_2(1 - p_1) \tag{6}$$

Suppose that the presence of each type of parasite reduces the fitness of each type of host, each host increases the fitness of each parasite, and there is competition between hosts of the same type and parasites of the same type. In this case, we can let $p_1 = p$ and $h_1 = h$ and write:

$$f_1 = 1 - ap - c(1 - p) - eh \tag{7}$$

$$f_2 = 1 - a(1 - p) - cp - e(1 - h) \tag{8}$$

$$g_1 = bh + d(1 - h) - kp \tag{9}$$

$$g_2 = b(1 - h) + dh - k(1 - p) \tag{10}$$

where a represents the cost to hosts of parasites of the same type, b the benefit to parasites of hosts of the same type, c the cost to hosts of parasites of the alternative type, d the benefit to parasites of hosts of the alternative type, e the competition between hosts of the same type and k the competition between parasites of the same type.

The Jacobian matrix of this system (whether continuous or discrete) is:

$$J = \begin{pmatrix} 1 - \frac{e}{2} & \frac{3}{4}(c - a) \\ \frac{3}{4}(b - d) & 1 - \frac{k}{2} \end{pmatrix} \tag{11}$$

The eigenvalues of this system are therefore given by:

$$\lambda = \frac{T\sqrt{T^2 - 4D}}{2} \tag{12}$$

where:

$$T = 2 - \frac{e+k}{2} \tag{13}$$

$$D = (\frac{e}{2} - 1)(\frac{k}{2} - 1) - \frac{9}{16}(b-d)(c-a) \tag{14}$$

In figure 5, we use the following parameter values:

Sub-Panel	(b)	(c)
a	0.4	0.5
b	0.9	0.5
c	0.9	0.4
d	0.4	0.4
e	3	5
k	3	5

In sub-panel (b), this gives:

$$\lambda_1 = -\frac{1}{8} \tag{15}$$

$$\lambda_2 = -\frac{7}{8} \tag{16}$$

This clearly lies in the red region, and we can see that the dynamics tend to a stable equilibrium in both the discrete and continuous models.

In sub-panel (c), on the other hand, we get:

$$\lambda_1 = -1.5 + \frac{3}{40}i \tag{17}$$

$$\lambda_2 = -1.5 - \frac{3}{40}i \tag{18}$$

This clearly lies in the blue region, and we can see that the dynamics tend to a stable equilibrium in the continuous case but are unstable in the discrete case.

3 Deterministic/Stochastic Model

3.1 The Model

Figure 6 in the main text looks at the effects of incorporating stochasticity into a deterministic model. The time series shown in this figure are generated using the following model.

Consider two sub-populations of hosts interacting with two sub-populations of parasites, under a matching-allele model (so parasite type 1 can only infect host type 1 and parasite type 2 can only infect host type 2). Let S_i denote the density of susceptible hosts of type i and I_i denote the density of infected hosts of type i (and hence also the density of type i parasites). We model this situation using the following system:

$$\frac{\mathrm{d}S_1}{\mathrm{d}t} = b(S_1 + I_1) - \beta S_1 I_1 \tag{19}$$

$$\frac{\mathrm{d}S_2}{\mathrm{d}t} = b(S_2 + I_2) - \beta S_2 I_2 \tag{20}$$

$$\frac{\mathrm{d}I_1}{\mathrm{d}t} = \beta S_1 I_1 - \alpha I_1 \tag{21}$$

$$\frac{\mathrm{d}I_2}{\mathrm{d}t} = \beta S_2 I_2 - \alpha I_2 \tag{22}$$

where b is the host birth rate, β is the transmission rate and α is the disease-induced mortality.

In figure 6, we consider the case where $b = 1$, $\beta = 10$ and $\alpha = 4$.

3.2 The Deterministic Model

As it stands, it is possible to determine the stability of this system. The endemic equilibrium is given by:

$$S_1^* = \frac{\alpha}{\beta} \tag{23}$$

$$S_2^* = \frac{\alpha}{\beta} \tag{24}$$

$$I_1^* = \frac{b\alpha}{\beta(\alpha - b)} \tag{25}$$

$$I_2^* = \frac{b\alpha}{\beta(\alpha - b)} \tag{26}$$

The Jacobian matrix of this system is given by:

$$J = \begin{pmatrix} -\beta I_1^* + b & 0 & -\beta S_1^* + b & 0 \\ 0 & -\beta I_2^* + b & 0 & -\beta S_2^* + b \\ \beta I_1^* & 0 & \beta S_1^* - \alpha & 0 \\ 0 & \beta I_2^* & 0 & \beta S_2^* - \alpha \end{pmatrix} \tag{27}$$

Substituting in the expressions for the endemic equilibrium, we can then calculate the eigenvalues of this matrix.

$$\lambda_1 = \lambda_2 = \frac{-b^2 + \sqrt{-4\alpha^3 b + 8\alpha^2 b^2 - 4\alpha b^3 + b^4}}{2(\alpha - b)} \tag{28}$$

$$\lambda_3 = \lambda_3 = -\frac{b^2 + \sqrt{-4\alpha^3 b + 8\alpha^2 b^2 - 4\alpha b^3 + b^4}}{2(\alpha - b)} \tag{29}$$

In the case where $b = 1$, $\beta = 10$ and $\alpha = 4$, as in figure 6, we get:

$$\lambda_1 = \lambda_2 = \frac{-1 + i\sqrt{143}}{6} \tag{30}$$

$$\lambda_3 = \lambda_4 = \frac{-1 - i\sqrt{143}}{6} \tag{31}$$

This is a continuous model and $\mathrm{Re}(\lambda) < 0$ for all eigenvalues, and so this endemic equilibrium is stable. This concurs with figure 6(a), where we can see damped oscillations.

3.3 The Stochastic Model

So far, we have considered a deterministic model. We introduce stochasticity by incorporating small, random changes in both host and parasite densities at fixed time intervals.

We can see in figure 6(b) that this causes the system to lose its stability. The damped oscillations in the deterministic case are now forced so that cycles are maintained indefinitely.
